# Synergizing Electrons and Photons in Motion: Continuous-Flow Implementation in Electro- and Photocatalyzed C–H Functionalization

**DOI:** 10.1021/jacsau.5c01706

**Published:** 2026-01-28

**Authors:** Sven Erik Peters, Tristan von Münchow, Lutz Ackermann

**Affiliations:** Wöhler Research Institute for Sustainable Chemistry (WISCh), 9375 Georg-August-Universität Göttingen, 37077 Göttingen, Germany

**Keywords:** Continuous-Flow, C—H Functionalization, Electrochemistry, Enantioselective Catalysis, Photochemistry, Photoelectrocatalysis

## Abstract

Continuous-flow technology has emerged as a powerful platform for resource-economical molecular synthesis, thereby addressing key limitations of conventional batch processes through unparalleled control of temperature and residence time as well as improved heat and mass transfer. Especially, the application of flow chemistry to C—H functionalization bears unique potential. By leveraging otherwise inert C—H bonds as latent functional groups, the step- and atom-economical access to value-added molecular architectures from abundant and readily available starting materials is facilitated. Thereby, flow reactor technology enables superior heat and mass transfer, accelerated kinetics, and direct scalability, promoting operationally simple, safe, and sustainable transformations. Continuous-flow has proven enabling in photo- and electrocatalysis as well as their synergistic merger in photoelectrochemical catalysis. In addition, these strategies unlock the use of earth-abundant catalysts and renewable solvents, while streamlining molecular synthesis.

## Graphical abstract

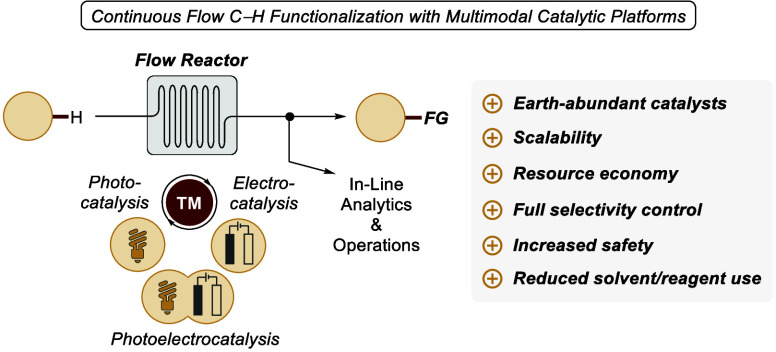

## Introduction

The continuous pursuit of more efficient and selective, molecular syntheses has, in recent decades, driven chemists to reconsider not only individual transformations, but also the platforms on which they are carried out.^1^ Thus, continuous-flow chemistry has emerged as a uniquely powerful technology, fundamentally redefining how reactions are executed, controlled, and scaled.^2–6^ In contrast to conventional batch processing, flow operation allows for precise and rapid tuning of key parameters, such as temperature, residence time, mixing, and stoichiometry.^1,2,7^ Thus, turning reactivity from an empirical compromise into an engineered variable. This level of control often translates into higher yields, improved selectivity, and significantly safer operation.^1,2,7–9^

Advances in reactor design are central to these developments: small reaction volumes and greatly increased surface-to-volume ratios afford exceptional heat- and mass transfer performance, enabling rapid kinetics, precise thermal management, and the safe handling of short-lived or hazardous intermediates.^2,7,10–16^ The frequent, inherent reproducibility of transformations in continuous-flow facilitates systematic optimization and seamless translation from milligram-scale discovery to multikilogram manufacturing without tedious process development.^12,17–19^ Hence, closing the long-standing gap between academic innovation and industrial application.^12,17^

At the same time, continuous-flow approaches align seamlessly with the principles of green chemistry.^1,20,21^ Operating in a confined, continuous environment minimizes solvent and reagent consumption, improves energy efficiency, and mitigates risk through inherently small, controlled reaction volumes.^1^ When realized with earth-abundant catalysts, renewable solvents, and recoverable heterogeneous systems, these features materially reduce the environmental footprint of complex molecule synthesis. Looking ahead, the integration of modular reactor architectures with in-line analytics, automation, and machine learning-driven optimization holds the promise of elevating continuous-flow chemistry from an enabling platform to an autonomous discovery and manufacturing tool — accelerating reaction innovation, shortening development timelines, and tightly connecting mechanistic understanding, process control, and scalable synthesis.^22–27^

Direct C–H bond functionalization ranks among the most atom- and step-economical strategies in molecular synthesis, enabling the rapid assembly of structurally complex products directly from simple, readily available feedstocks — without recourse to prefunctionalized substrates.^28–30^ However, despite its conceptual elegance and broad applicability, conventional batch-based approaches often suffer from intrinsic shortcomings: inefficient mixing and mass transfer, inadequate thermal management, and pronounced scale-up difficulties.^31^ Continuous-flow technology effectively addresses these challenges (Scheme 1). By delivering precise control over residence time, temperature, and reagent stoichiometry — in conjunction with superior heat and mass transfer — flow reactors unlock the finely tuned conditions required for efficient and selective catalytic C–H functionalization.^2,32^

**Scheme 1:**
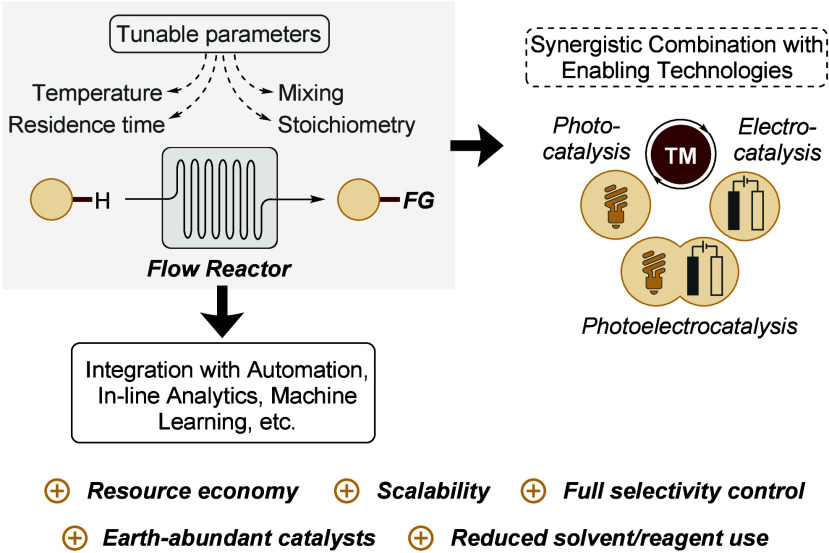
Flow Experimentation as an Enabling Tool in Molecular Synthesis

Early examples of organometallic C–H activation in flow include a ruthenium-catalyzed *ortho*-arylation of 2-phenylpyridine,^33^ followed by distinct thermal homogeneous^34–40^ and heterogeneous^41–49^ C—H functionalizations. Notably, thermal flow inner-sphere C—H activations had been limited to the use of precious and expensive 4d and 5d transition metals, including palladium, ruthenium, rhodium, and iridium. In sharp contrast, major advance was represented by making earth-abundant 3d transition metals viable for challenging flow-C—H activations. Thus, in 2017, manganese­(I)-catalyzed hydroarylations were achieved without precautions for an inert atmosphere relying on a residence time of under 20 min— dramatically faster than conventional batch protocols that typically require several hours (Scheme 2).^50^ This strategy delivered allylic carbonates and ethers in a step-economical fashion, with ample scope and excellent chemo-, and regioselectivity (Scheme 2A). Operational simplicity and scalability (Scheme 2B), including facile in-line catalyst separation and recycling represent further beneficial features (Scheme 2C).^26,51^

**Scheme 2:**
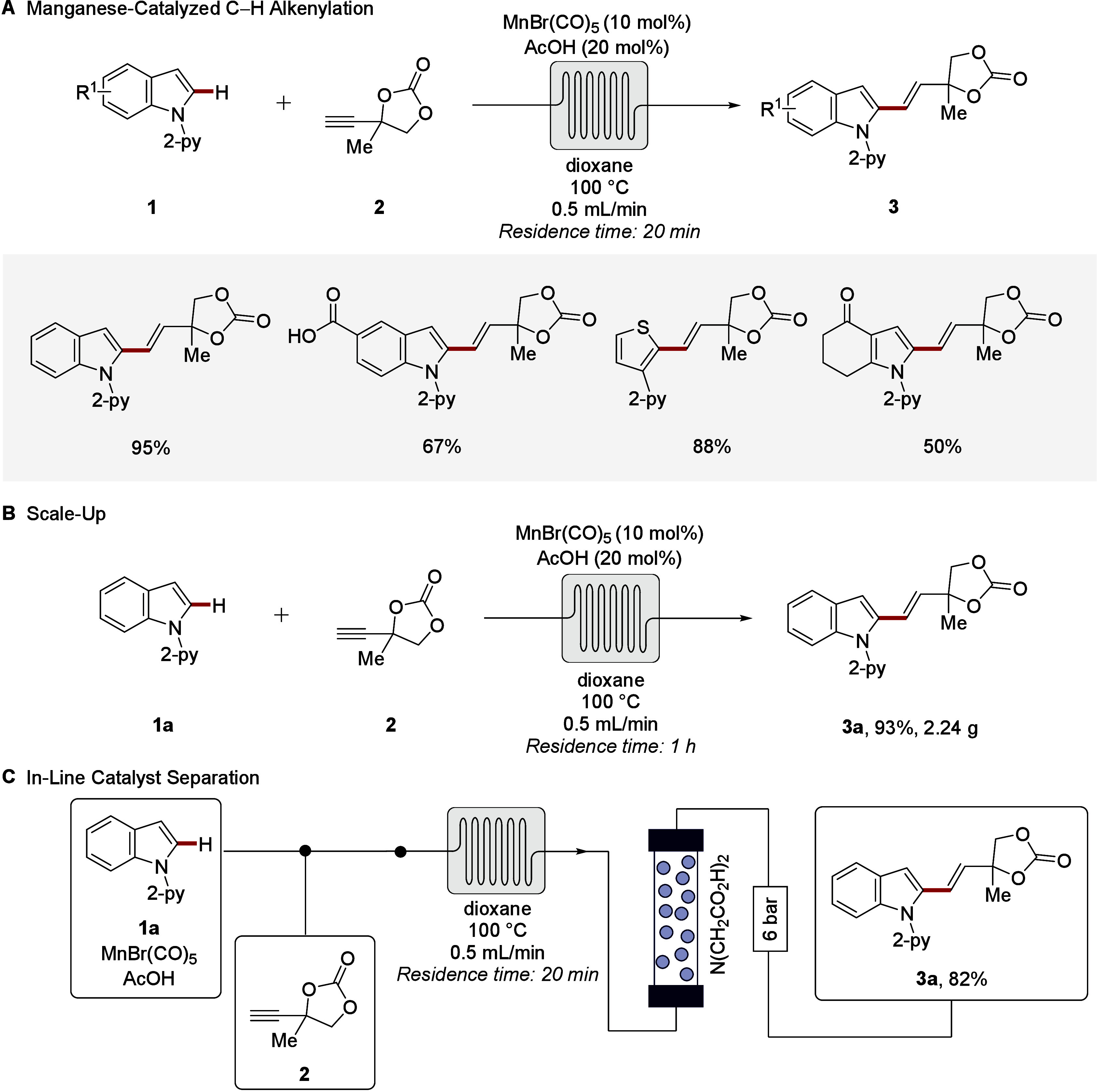
Thermal Manganese­(I)-Catalyzed C–H Activation in Continuous-Flow Enabling Efficient Hydroarylations

Further, thermal flow manganese-catalyzed C–H arylations proved viable on pyridines by means of low-valent metal catalysis via single-pass continuous-flow technology (Scheme 3A).^52^ Thus, inexpensive most user-friendly MnCl_2_ enabled gram-scale C–H arylations within 100 min (Scheme 3B). An additional asset of the flow approach is constituted by the safe and efficient handling of rather reactive Grignard reagents on scale.^53^ The flow strategy set the stage for a telescoped 2-fold C—H activation manifold, both operating with benign 3d transition metals, namely manganese and iron (Scheme 3C).

**Scheme 3:**
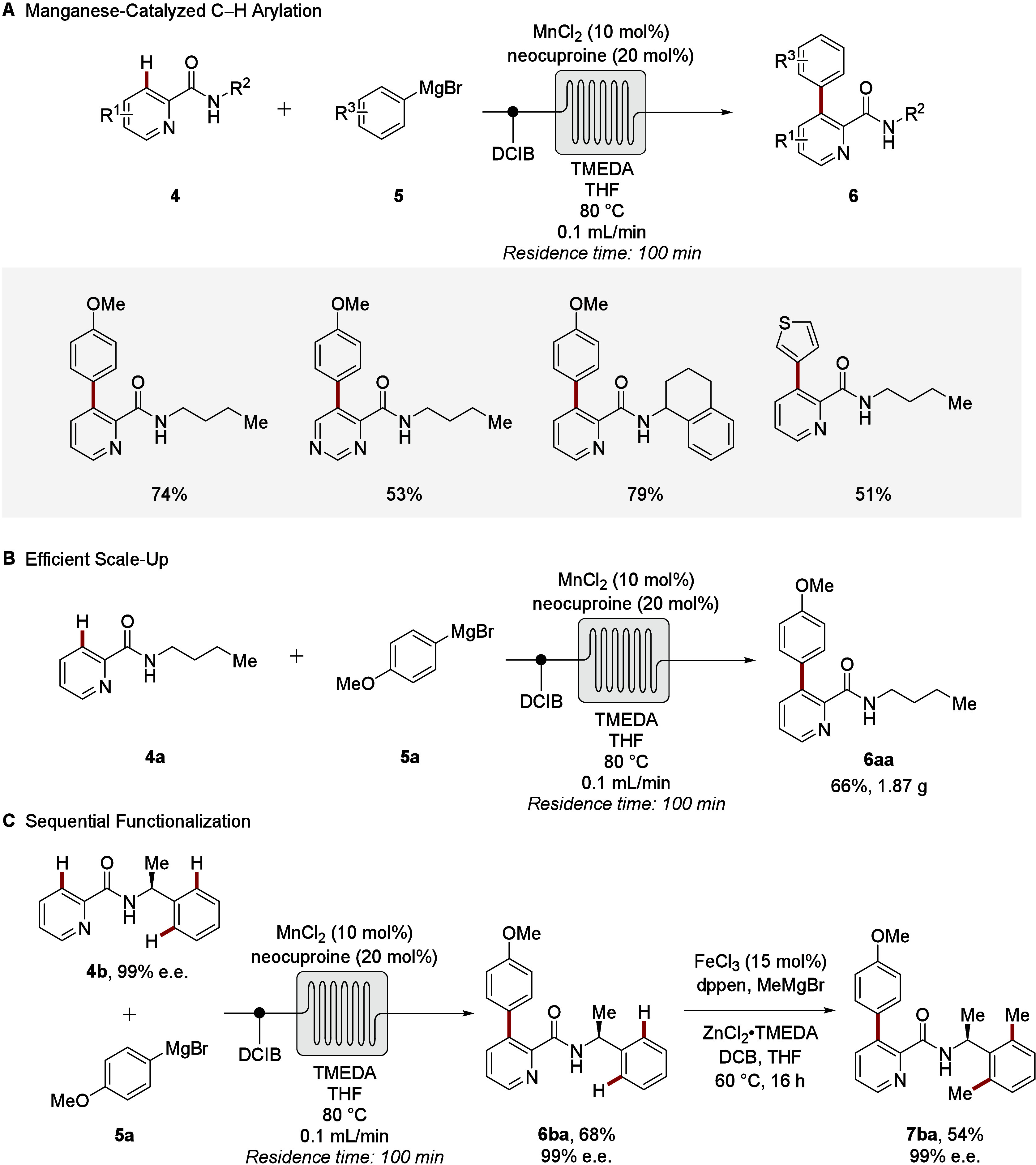
Manganese­(II/III/I)-Catalyzed C–H Arylations of Heteroarenes **4** in Continuous-Flow

Despite of these indisputable advances in thermal flow catalysis,^32^ photo- and electrocatalysis hold thus far unmatched potential by harnessing photons and electrons as traceless reagents for more efficient bond formation toward an effective energy transition. Hence, we herein provide an overview of recent developments in flow C–H functionalizations enabled by photochemistry, electrochemistry, and synergistic photoelectrochemistry up to December 2025.

## Photocatalytic C–H Activation in Flow

Photochemistry has witnessed tremendous recent momentum with the prospect of employing light as a traceless reagent. By exploiting the unique reactivity of electronically excited states, photochemistry can unlock transformations that are often inaccessible under purely thermal conditions.^54–59^ However, conventional batch photochemistry faces intrinsic limitations primarily due to the light attenuation effect in terms of the Bouguer-Lambert–Beer law. Thus, inefficient photon penetration, nonuniform irradiation, and localized overheating can undermine efficiency, reproducibility, and selectivity, especially for the transition by the practitioners on scale.^60,61^ Continuous-flow photochemistry has the power to overcome these challenges. Owing to their high surface-area-to-volume ratios, the small depth, and facile heat dissipation, flow reactors ensure uniform irradiation, efficient photon utilization, and precise control over reaction temperature.^62–64^ This level of control in conjunction with the residence time is especially critical where the fleeting nature of key intermediates demands a meticulously tuned balance between reactivity and selectivity.^9,62–64^ Crucially, continuous-flow enables thus the streamlined upscaling of photochemical processes.^62–65^

To this end, Stephenson highlighted the scalability in photoredox catalysis by developing a low-path-length flow photoreactor for oxidative functionalizations of tetrahydroisoquinolines **8** with diverse nucleophiles, using a precious ruthenium dye (Scheme 4).^66^ Notably, the functionalization was viable within 30 s residence time, drastically improving the reaction rate as compared to established batch conditions. This study served as the blueprint for subsequent C—H functionalizations of activated amines.^67–71^

**Scheme 4:**
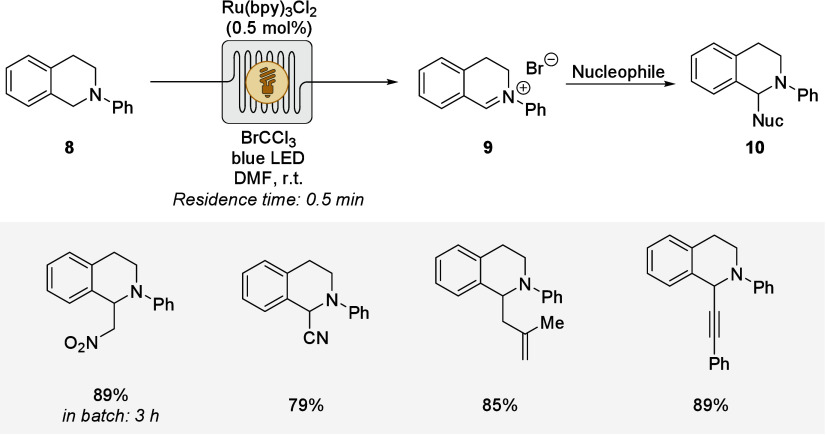
Oxidative Functionalization of Tetrahydroisoquinolines **8**

Further recent developments focused on controlled radical-based chemistry for efficient C­(sp^3^)—H functionalization. Thereby, Wu devised a photochemical strategy for the alkylation of unactivated C­(sp^3^)—H bonds, employing an organic photosensitizer and HCl as a hydrogen atom transfer (HAT) precatalyst (Scheme 5A).^72^ A microtubing reactor proved crucial for efficiently using volatile HCl, enabling the preparation of different drug compounds from readily available alkanes **11**. After initial reports on decatungstate photocatalysis under flow conditions,^73,74^ Noël and co-workers established the aerobic oxidation of activated and unactivated C­(sp^3^)—H bonds (Scheme 5B).^75^ While in batch full conversion was not achieved, the improved oxygen diffusion and irradiation under flow conditions led to more effective transformations. Hence, the selective oxidation of complex natural products, such as (—)-ambroxide and artemisinin, was accomplished. Subsequently, related strategies for productive C­(sp^3^)—H functionalization enabled acylation, amination, and arylation as well as heteroarylation in flow.^76–78^ Additionally, Ye disclosed a scalable flow approach for the preparation of γ-undecalactone — a fragrance molecule with peach aroma (Scheme 5C).^79^ By exploiting an organic dye, the targeted compound **18aa** was accessed through photoredox-catalyzed C­(sp^3^)—H alkylation, utilizing 1-octanol **11a** and methyl acrylate **17a** as abundant feedstocks.

**Scheme 5:**
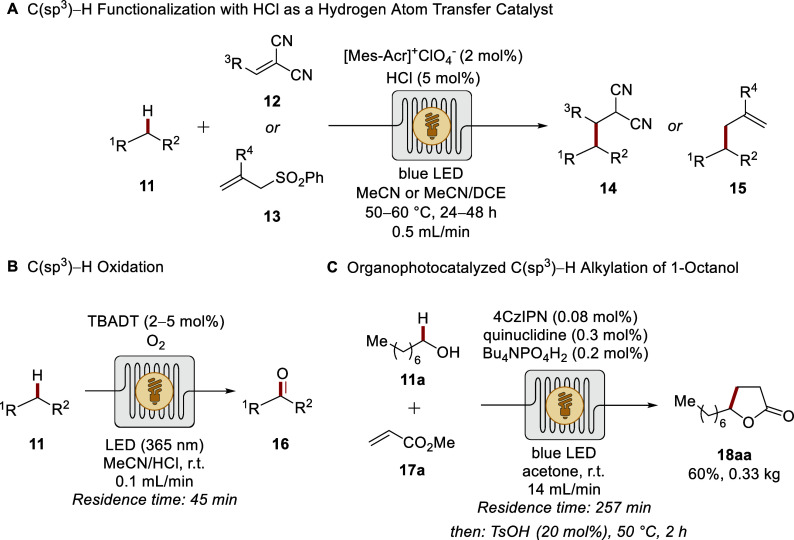
Photocatalytic C­(sp^3^)—H Functionalizations in Flow

On a different note, Stephenson devised a photocatalytic strategy for the trifluoromethylation of diverse (hetero)­arenes with the aid of pyridine *N*-oxide, trifluoroacetic anhydride, and a precious metal-based photoredox catalyst (Scheme 6).^80^ The most-user-friendly strategy was exploited for a successful kilogram scale-up by continuous-flow experimentation, affording pyrrole derivative **20**.

**Scheme 6:**
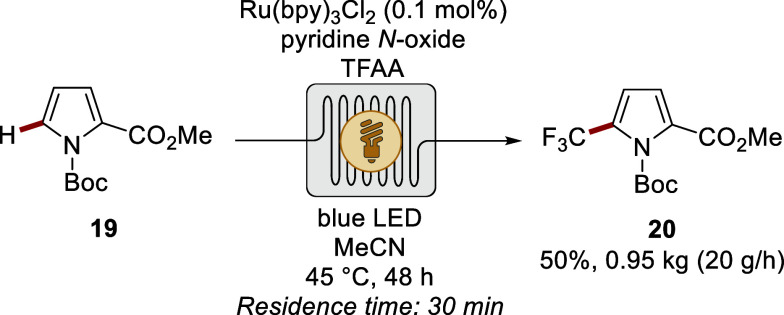
Photocatalytic Trifluoromethylation of Pyrrole Derivative **19** in Continuous-Flow

In contrast, the cost-effective 3d transition metal manganese was successfully employed for continuous photoflow C–H arylations in 2018. The manganese-catalyzed photoflow C–H arylation proved viable under visible light irradiation at room temperature (Scheme 7).^81^ Efficient C–H arylations were accomplished with a residence time of 30 min, using diazonium salts as productive aryl radical precursors (Scheme 7A). Salient features of the flow-photocatalysis include ample scope and ease of scale-up (Scheme 7B). Thus, the visible-light-induced C–H arylation of benzene afforded biaryl product **23aa** in excellent yield within 60 min in flow, whereas an analogous batch reaction delivered only 25% yield. These findings reflect the augmented efficacy for energy- and mass transfer. The applicability was further showcased by the functionalization of biomass-derived furfural **22b**; thus, setting the stage for an elegant route to a key intermediate for Dantrolene **24bb**, a clinically relevant drug for the treatment of malignant hyperthermia (Scheme 7C).

**Scheme 7:**
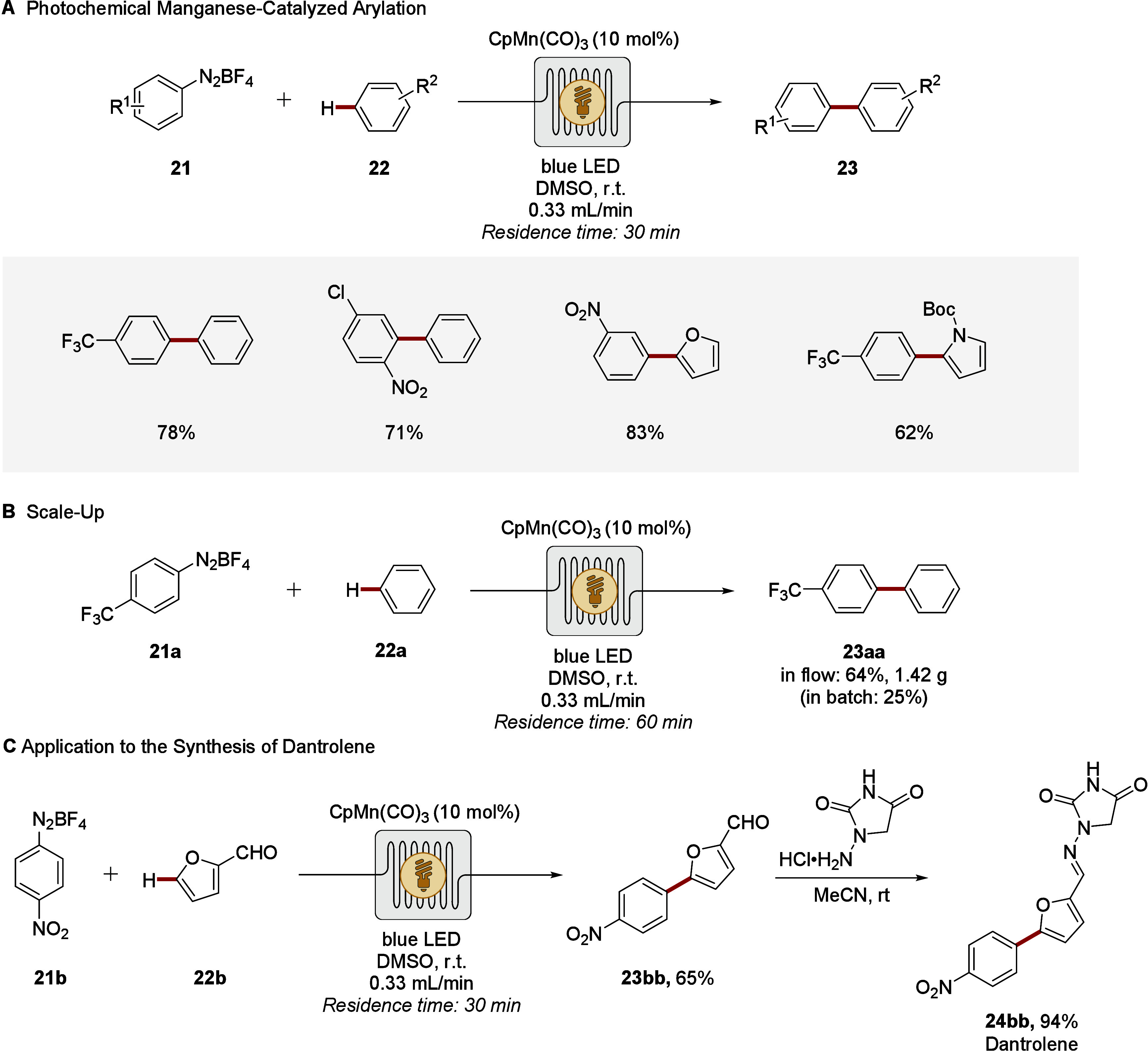
Manganese-Catalyzed (Hetero)­arene C–H Arylation Implementing Visible Light Photo-flow

The power of inner-sphere C—H activation by photoredox-catalysis in flow was first showcased by Noël and Van der Eycken (Scheme 8).^82^ Thus, efficient C2 acylation of the indole scaffold was accomplished using aromatic and aliphatic aldehydes. Thereby, a precious metal-based photoredox catalyst ensures the formation of acyl radicals and the oxidation of the proposed intermediate **Pd1** *via* single-electron transfer (SET) for effective reductive elimination. A drastic increase in reaction rate and efficiency as well as a decreased catalyst loading underlined the potential of flow experimentation in this instance. Nevertheless, the usage of noble metal catalysts compromised sustainability.

**Scheme 8:**
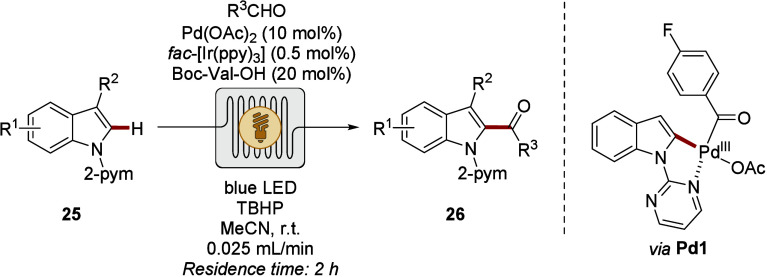
Palladium-Catalyzed C—H Acylation of Indoles **25** by Flow Photoredoxcatalysis

Most marketed drugs and crop-protecting agents are chiral. Hence, there is continued strong, and yet largely unmet demand for enantioselective C—H activations. To this end, the implementation of enantioselective catalysis is of considerable topical interest to enable streamlined and selective access to three dimensionality.^83–87^ Specifically, high-valent cobalt compounds have been recognized as sustainable and powerful tools for enantioselective C—H activations, with key contributions by Ackermann,^88,89^ Cramer,^90^ Matsunaga,^91^ and subsequently Shi.^92^ Inspired by findings on electro- and photocatalytic cobalt-catalyzed C—H activations by Ackermann and Sundararaju,^93–96^ an unprecedented photoredox-enabled enantioselective cobalt-catalyzed C–H activation was devised, demonstrating excellent compatibility with flow conditions (Scheme 9).^97^ The use of organic dyes in place of precious-metal photocatalysts, combined with the absence of sacrificial metal-based oxidants mirrored the sustainable nature of this approach. The operational robustness was demonstrated by a gram-scale synthesis in flow at reduced catalyst loading, highlighting the scalability of cobaltaphotoredox catalysis under flow conditions (Scheme 9A). The dual catalytic manifold also enabled the regio- and stereoselective dearomatization of indoles to access the chiral products **28** with high chemical yield and enantioselectivities up to 99% ee. The strategy proved equally effective for constructing both central and axial chirality, while accommodating a diverse substrate scope, including unactivated alkenes (Scheme 9B and Scheme 9C). Concurrently, related approaches were disclosed by Shi and Sundararaju employing batch reaction conditions.^98,99^

**Scheme 9:**
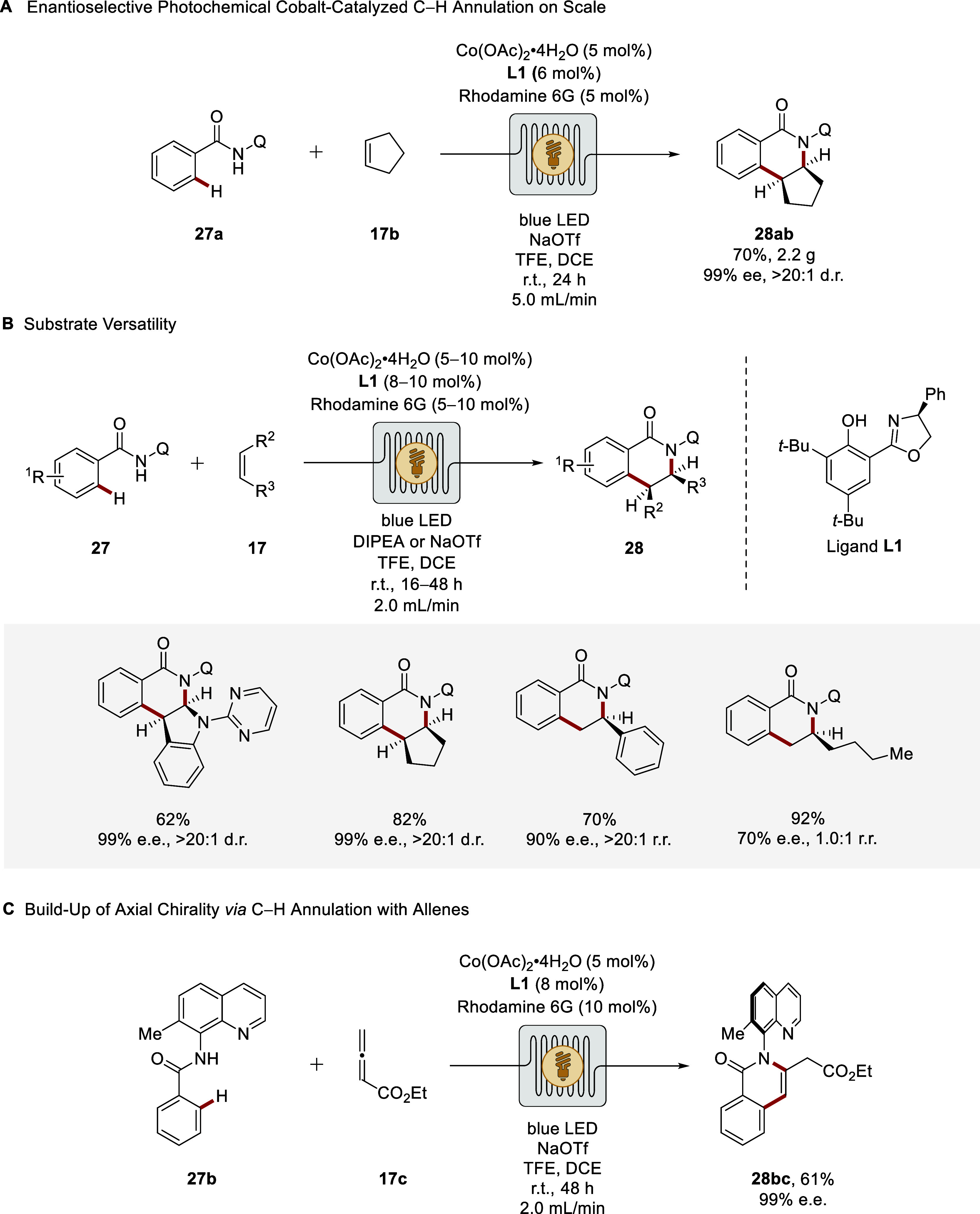
Enantioselective Cobaltaphotoredox-Catalyzed C–H Activation in Flow

The *modus operandi* relies on a cobalt­(II/III/I) regime (Scheme 10).^100–107^ Thereby, initial SET mediated by the organic dye sets the stage for a base-assisted C–H activation. The resulting cyclometalated intermediate **Co3** was isolated and fully characterized by leveraging a stabilizing ligand. Subsequent coordination of the olefin followed by migratory insertion and reductive elimination furnishes the desired product **28ad**, whereby the concomitantly formed cobalt­(I) species is reoxidized through sequential SET processes.

**Scheme 10:**
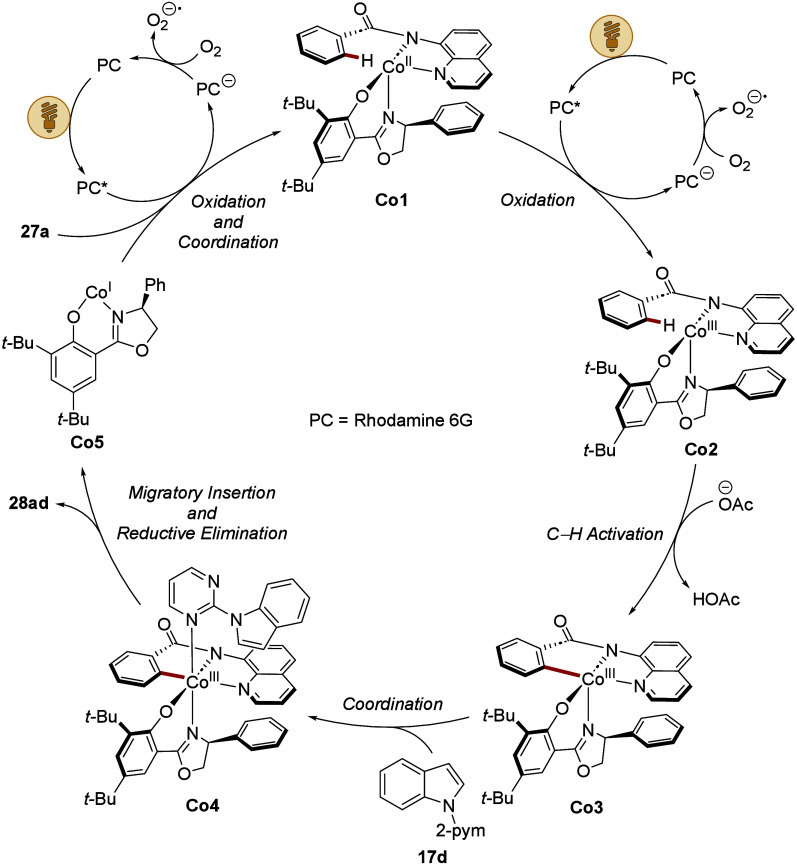
Proposed Mechanism for the Enantioselective Cobaltaphotoredox-Catalyzed C–H Activation

Very recently, the versatility of merging photoredox catalysis with enantioselective cobalt catalysis was further demonstrated by the kinetic resolution of *ortho*- and pseudodisubstituted multichiral [2.2]­paracyclophanes (PCPs) (Scheme 11), relying on a related mode of operation.^108,109^ These sterically congested and stereochemically rich scaffolds are of considerable value in asymmetric catalysis and functional materials, yet remain challenging to access by conventional C–H activation strategies. By integrating precise photon flux control with the inherent stereocontrol of cobalt catalysis in flow, the construction of planar- and central-chiral PCP derivatives was realized with exceptional enantioselectivity (>99% e.e.) and high diastereoselectivity (>20:1 d.r.), while simultaneously recovering the unreacted enantiomer in high optical purity (Scheme 11A and Scheme 11B).^108^ The approach proved amenable to gram-scale synthesis without erosion of selectivity, and the products could be readily diversified into PCP-based ligands for asymmetric catalysis, further enriching their synthetic utility.

**Scheme 11:**
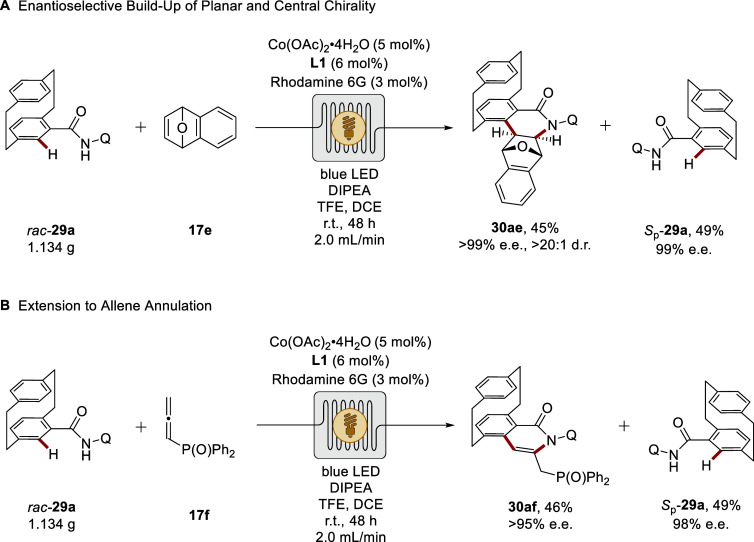
Stereocontrolled Construction of [2.2]­Paracyclophanes **30** in Flow

## Electrocatalytic C–H Functionalization in Flow

Electrochemistry bears a rich history, originating from the fundamental findings by Faraday and Kolbe regarding the decarboxylative dimerization of carboxylic acids.^110,111^ In recent years, it has re-emerged as a sustainable tool in modern molecular syntheses, providing a green alternative to stoichiometric redox reagents by proton and electron transfer as traceless reagents.^112–119^ Electrochemical transformations allow precise control of chemo-selectivity by dialing in the exact redox potentials, enabling selective oxidation or reduction processes under exceedingly mild conditions.^120–122^ Additionally, for electrooxidatively driven functionalizations, molecular hydrogen is formed through hydrogen evolution reaction (HER) as a byproduct of utmost relevance.^123–125^

Conventional batch electrochemistry, however, is often hampered by restricted mass transport to the electrodes, and hence challenges in scale-up.^126,127^ Continuous-flow electrochemistry addresses these challenges by optimizing the interaction between the reaction medium and electrode surfaces.^128–133^ Flow reactors provide a high surface-area-to-volume ratio, which promotes uniform current distribution and efficient electron transfer. This improved mass and electron transfer enhances reaction efficiency, minimizes overoxidation or -reduction, and allows reactions to be conducted at higher concentrations with improved reproducibility. Simultaneously, the usually small interelectrode gap diminishes the ohmic drop, allowing for a substantially decreased amount of required supporting electrolyte. Continuous-flow setups also facilitate scalability and improved safety, making them particularly attractive for industrial applications.^128–133^

Thereby, recent studies showcased the potential of electrocatalysis under continuous-flow to be an efficient tool in C—H functionalization *via* SET-enabled radical pathways. Wirth and Xu established a strategy for the synthesis of benzothiazoles *via* dehydrogenative, intramolecular C—S bond formation in continuous-flow.^134^ Thereby, the successful omission of supporting electrolyte, a gram scale-up, and an elevated reaction performance underpinned the strength of flow chemistry compared to its batch analogue (Scheme 12A). Subsequently, Xu devised a variety of electrochemical manifolds for the direct C—H phosphorylation, hydroxylation, oxidation, and amination, whereby exceptional residence times as low as 22 s and broad scale-ups up to 204 g proved the advantageous reaction control (Scheme 12B).^135–139^ Further examples by Waldvogel and Noël demonstrated the thriving application of continuous-flow in C—H functionalization, regarding the dehydrogenative preparation of a bisphenol and the azolation as well as hydroxylation of arenes.^140–142^

**Scheme 12:**
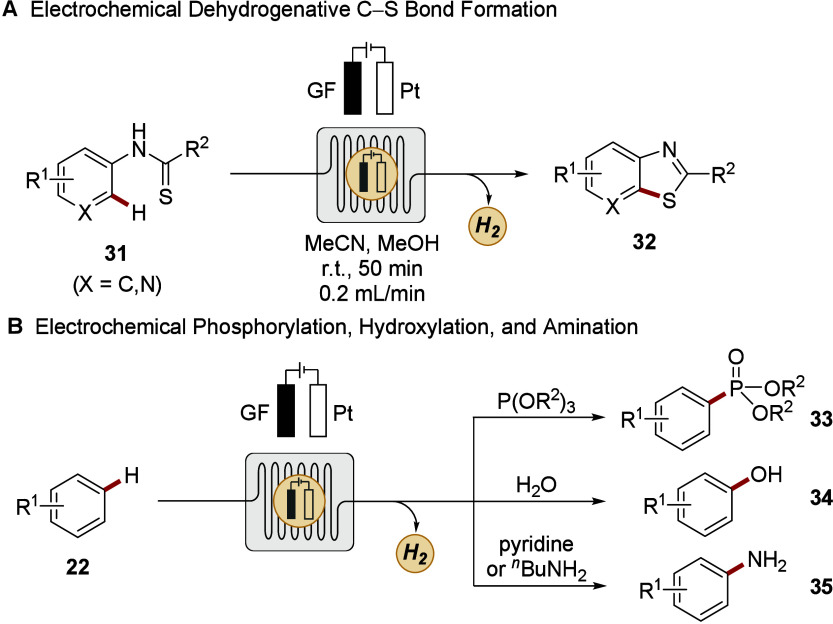
Electrocatalytic C—H Functionalizations under Continuous-Flow Conditions

In sharp contrast, recent studies have highlighted the synergy between flow electrochemistry and inner-sphere C–H activation to enable tailored and traceless transfers of protons and electrons. Ackermann and co-workers devised a rhodium-catalyzed electrochemical C–H annulation in flow (Scheme 13).^143^ A modular flow reactor equipped with a porous graphite felt anode was designed. Thus, efficient mass and electron transfer were ensured, fostered by a meshed thin PTFE plate as turbulence promoter. This system enabled both inter- and intramolecular C–H/N–H functionalization with excellent selectivity and versatile scope. Noteworthily, efficient scale-up proved viable. Additionally, the reaction could be monitored in real-time using online-flow NMR spectroscopy, supporting the decisive role of anodic oxidation for product formation. Further studies, involving the isolation and characterization of rhodium­(III)-heptacycle **Rh1** as a key intermediate, uncovered an oxidation-induced reductive elimination regime as part of a Rh­(III/IV/II) catalytic cycle.^144^ This study mirrored the viability of efficient and scalable C–H activation *via* metallaelectrocatalysis under flow conditions while simultaneously enabling the elucidation of mechanistic intricacies through online analytics.

**Scheme 13:**
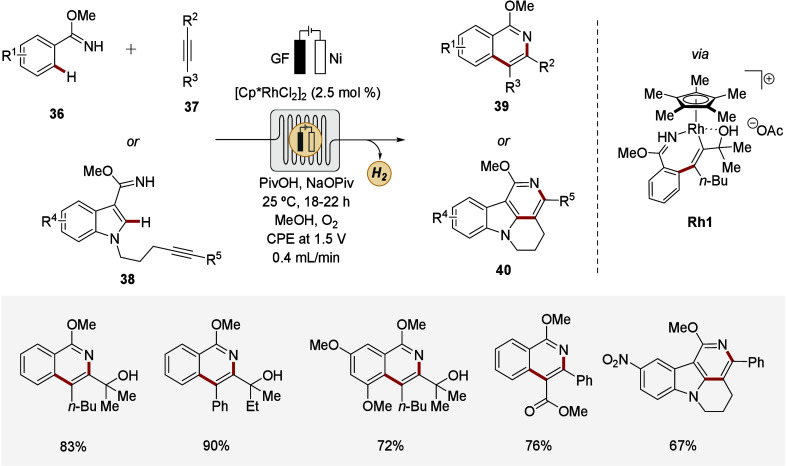
Flow Rhodaelectro-catalyzed Alkyne Annulations

Subsequently, Ackermann devised a strategy for the expedient access to seven-membered azepino­[3,2,1-*hi*]­indoles through dehydrogenative rhodaelectro-catalyzed C—H/N—H annulation in flow.^145^ Recently, Shi reported a related approach for accessing 1,2-benzothiazines.^146^

Motivated by elaborate studies on utilizing electricity *in lieu* of stochiometric oxidants for resource-economical cobalt-catalyzed C—H activation,^101,113,147,148^ Ackermann reported the first merger with enantioselective catalysis.^93^ Thereby, high enantioselectivities were obtained under galvanostatic control without electrode compartment separation. Subsequently, this strategy was successfully translated to flow conditions, enabling the synthesis of C–N axially chiral compounds **28** *via* allene C–H annulation.^102^ The approach allowed atroposelective access to axial chiral isoquinolinones **28** in high yields with excellent enantioselectivities and was compatible with complex bioactive molecules and drug candidates (Scheme 14A). The cyclometalated cobalt­(III) species **Co6** was isolated as a potential intermediate mimic. In conjunction with further experiments, involving, among others, cyclic voltammetry studies, a cobalt­(II/III/I) regime was unraveled (Scheme 10), ensuring catalytic turnover *via* anodic oxidation. The flow electrolysis setup did not require supporting electrolytes and enabled efficient scalability, as demonstrated by decagram-scale reactions with maintained high stereocontrol. The products could also be further transformed into phosphine ligands with axial chiral backbone, illustrating the synthetic versatility of the approach (Scheme 14B).

**Scheme 14:**
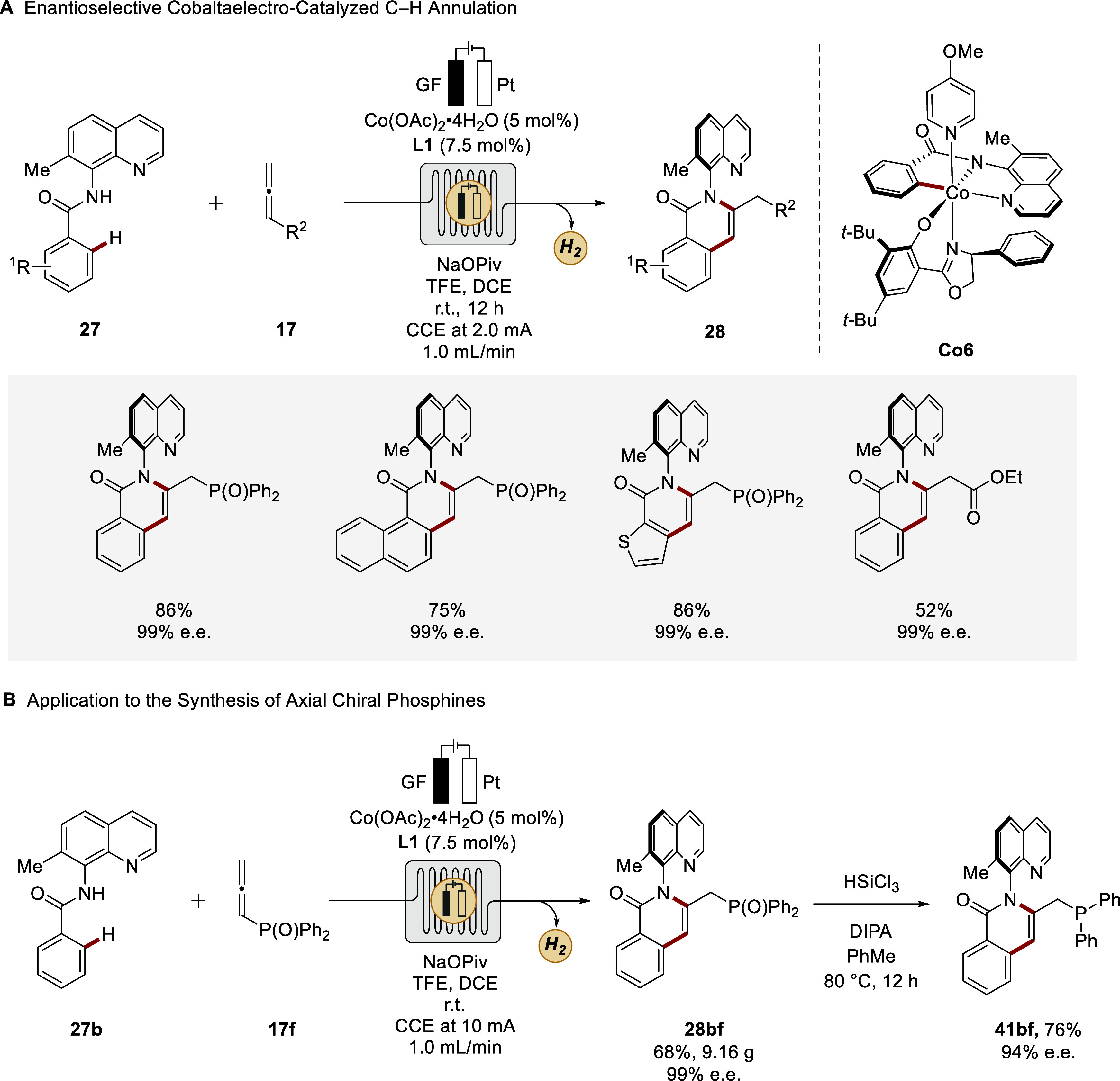
Atroposelective Cobaltaelectro-catalyzed C–H Annulations with Allenes

## Photoelectrocatalytic C–H Functionalization in Flow

The combination of photochemistry and electrochemistry in continuous-flow systems offers a powerful strategy for modern organic synthesis, leveraging the tailored synergy of photons and electrons for transformations that are difficult under conventional batch conditions.^149–151^ In such setups, light and electricity act collaboratively to activate chemical bonds in a selective fashion, providing controlled redox input and energy with high spatial and temporal precision. Continuous-flow operation enhances these advantages by ensuring uniform light penetration, efficient electron transfer, and rapid mass transport, collectively improving reaction efficiency, selectivity, and scalability.^61,62,131,152^ Further, flow chemistry allows for spatial separation of an ongoing transformation.^2^ Hence, distinct operations can be conducted sequentially in a linear, continuous arrangement, setting the stage for elegant reaction design with decoupled photo- and electrochemical manipulations.^26^

In 2020, Ackermann demonstrated the potential of this approach for C–H functionalization (Scheme 15A).^153^ They reported a photoelectro-catalyzed, undirected C–H trifluoromethylation of arenes **22**, merging photoredox catalysis with electrochemical oxidation in a flow setup. Trifluoromethyl radicals were generated from the Langlois reagent (CF_3_SO_2_Na) **42** under exceedingly mild, oxidant-free conditions through anodic regeneration of the ground state photocatalyst (Scheme 15B). A sequence consisting of the radical’s attack on **22b**, SET, and deprotonation furnishes the C–H functionalized product. The operational simplicity of the flow system, combined with broad versatility, highlighted its practical utility for scalable synthesis. The photoelectrocatalysis was performed in a modular electrochemical flow cell equipped with a graphite felt anode and a nickel cathode, followed by a looped transparent irradiation module. At a flow rate of 1.0 mL/min and a residence time of 6 min in the electrochemical cell, the trifluoromethylated product **43** was obtained efficiently. This configuration allowed spatial and temporal separation of electro-oxidation and photocatalysis, enabling both steps to occur concurrently yet independently; thus, minimizing overoxidation and side reactions. Intriguingly, online flow-NMR monitoring provided mechanistic insights, supporting the involvement of SET processes.

**Scheme 15:**
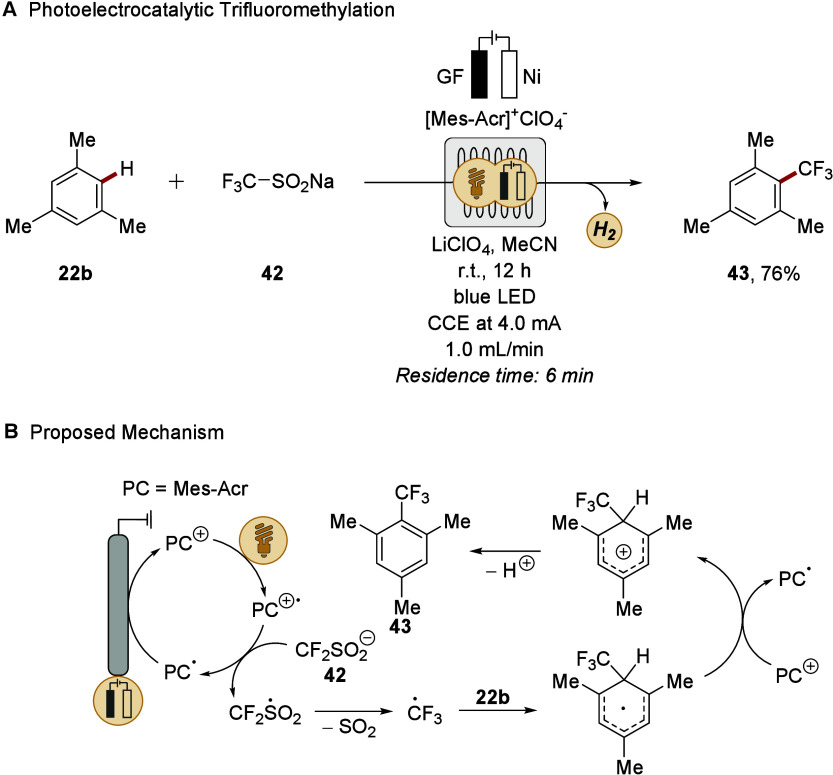
Photoelectrocatalytic Undirected C–H Trifluoromethylation in Flow

In response to this proof-of-concept study, other groups joined the endeavor of exploring the synergism between photons and electrons under flow conditions. Thereby, photochemical flow reactors cells are frequently applied in conjunction with batch electrochemical cells. For instance, Xu devised a strategy for the scalable preparation of novel 3,6-difunctionalized acridinium photocatalysts by leveraging a flow sequence, involving decoupled electro- and photochemical reactors (Scheme 16A).^154^ Crucially, a decagram synthesis of acridinium dye **45** was accomplished through a telescoped process involving two sequential C—H alkylations with an intermediate purification. Further, Xu devised a photoelectrocatalytic approach for dehydrogenative C­(sp^3^)—H arylation in the absence of external oxidants.^155^ Subsequently, Lambert reported a flow process involving an electrochemical cell and photochemical chambers for the gram-scale synthesis of phenol from benzene by photoelectrocatalytic C—H heterofunctionalization (Scheme 16B).^156^ Thereby, 2,3-dichloro-5,6-dicyano-1,4-benzoquinone (DDQ) served as a photoelectrocatalyst.

**Scheme 16:**
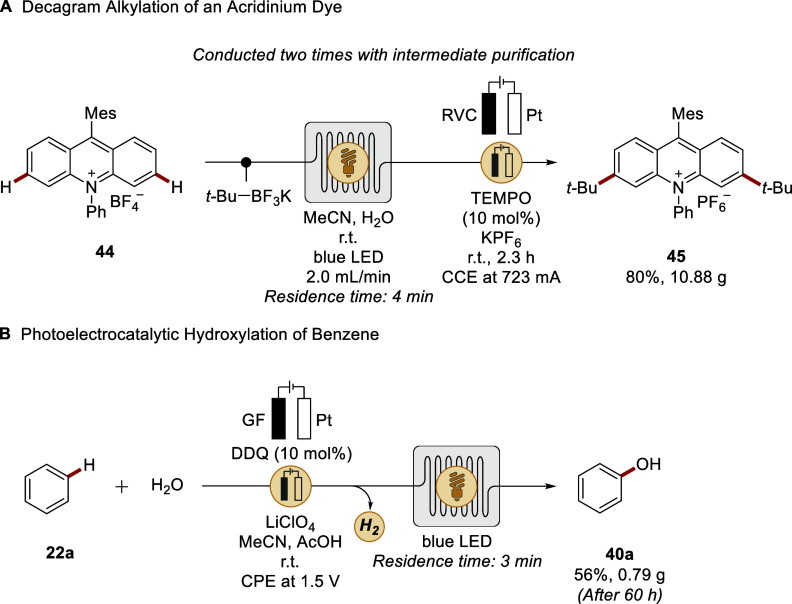
Photoelectrocatalytic C—H Functionalizations under Flow Conditions

In contrast, Reek and Noël developed a new reactor encompassing a transparent electrode for its application in C­(sp^3^)—N bond-forming heteroarylation reactions (Scheme 17).^157^ The mild and oxidant-free conditions set the stage for the functionalization of a broad set of saturated heterocyclic scaffolds, benefiting from the enhanced kinetics and productivities of flow chemistry. Furthermore, Guo and Xia took advantage of a flow platform for the scale-up of a photoelectrochemical oxidative C­(sp^3^)—H borylation, accessing valuable organoboron compounds from unactivated hydrocarbons.^158^

**Scheme 17:**
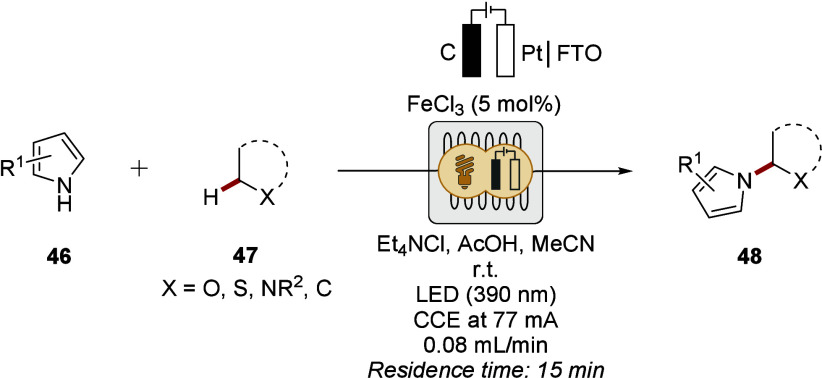
Photoelectrocatalytic Heteroarylations in Continuous-Flow

## Conclusion and Outlook

Mass and heat transfer often limits the effective scale-up of photo- and electrochemical transformations. Flow chemistry has the unique power to address these challenges by fundamentally reshaping how modern organic synthesis is designed, controlled, and scaled. By recasting the reactor as an engineered, tunable variable rather than a passive vessel, flow technology delivers unmatched control over residence time, temperature, mixing, photon flux, and electron flow. These attributes translate directly into higher yields, enhanced selectivity, improved safety, and straightforward scale-up. When coupled with contemporary catalytic manifolds, flow reactors have enabled dramatic improvements regarding photocatalytic and electrocatalytic C–H functionalization strategies, overcoming their batch limitations through uniform irradiation, efficient photon utilization, and superior mass transfer to well-defined electrode interfaces. Thereby, application to enantioselective platforms enabled the streamlined assembly of complex scaffolds. The synergistic merger of these activation modes in photoelectrochemical flow systems further expands the synthetic repertoire by providing orthogonal, temporally, and spatially resolvable inputs, generating reactive intermediates under mild, oxidant-free conditions.

Looking ahead, the most impactful developments will arise from combining modular, robust reactor hardware with in-line analytics, automation, and machine learning-guided optimization to enable real-time process understanding as well as autonomous optimization.^22–27^ Remaining challenges include standardizing modular hardware for multistep and multicatalytic sequences and enhancing the longevity and compatibility of flow systems regarding electro- and photochemical conditions. Given the practical importance of photo- and electrochemistry in continuous-flow for practitioners, platforms that unite organometallic C–H activation, photochemistry, electrochemistry, and photoelectrochemistry are expected to facilitate scale-up and accelerate fundamental discoveries.
